# Vaccine-Induced Th1-Type Response Protects against Invasive Group A *Streptococcus* Infection in the Absence of Opsonizing Antibodies

**DOI:** 10.1128/mBio.00122-20

**Published:** 2020-03-10

**Authors:** Tania Rivera-Hernandez, Mira Syahira Rhyme, Amanda J. Cork, Scott Jones, Celia Segui-Perez, Livia Brunner, Johanna Richter, Nikolai Petrovsky, Maria Lawrenz, David Goldblatt, Nicolas Collin, Mark J. Walker

**Affiliations:** a Australian Infectious Diseases Research Centre and School of Chemistry and Molecular Biosciences, The University of Queensland, St Lucia, QLD, Australia; b Cátedras CONACYT—Unidad de Investigación Médica en Inmunoquímica, Hospital de Especialidades del Centro Médico Nacional Siglo XXI, Instituto Mexicano del Seguro Social, Mexico City, Mexico; c Great Ormond Street Institute of Child Health Biomedical Research Centre, University College London, London, United Kingdom; d Vaccine Formulation Institute, Plan-Les-Ouates, Geneva, Switzerland; e Flinders University and Vaxine Pty. Ltd., SA, Australia; Max Planck Institute for Infection Biology

**Keywords:** group A *Streptococcus*, vaccines, adjuvants, invasive disease

## Abstract

Recent global advocacy efforts have highlighted the importance of development of a vaccine against group A *Streptococcus* (GAS). Combo5 is a non-M protein-based vaccine that provides protection against GAS skin infection in mice and reduces the severity of pharyngitis in nonhuman primates. However, Combo5 with the addition of aluminum hydroxide (alum) as an adjuvant failed to protect against invasive GAS infection of mice. Here, we show that formulation of Combo5 with adjuvants containing saponin QS21 significantly improves protective efficacy, even though all 7 adjuvants tested generated high antigen-specific IgG antibody titers, including alum. Detailed characterization of Combo5 formulated with SMQ adjuvant, a squalene-in-water emulsion containing a TLR4 agonist and QS21, showed significant differences from the results obtained with alum in IgG subclasses generated following immunization, with an absence of GAS opsonizing antibodies. SMQ, but not alum, generated strong interleukin-6 (IL-6), gamma interferon (IFN-γ), and tumor necrosis alpha (TNF-α) responses. This work highlights the importance of adjuvant selection for non-M protein-based GAS vaccines to optimize immune responses and protective efficacy.

## Abstract (precis)

Availability of a group A *Streptococcus* vaccine remains an unmet public health need. Here, we tested different adjuvant formulations to improve the protective efficacy of non-M protein vaccine Combo5 in an invasive disease model. We show that novel adjuvants can dramatically shape the type of immune response developed following immunization with Combo5 and significantly improve protection. In addition, protection afforded by Combo5 is not mediated by opsonizing antibodies, believed to be the main correlate of protection against GAS infections. Overall, this report highlights the importance of adjuvant selection in raising protective immune responses against GAS invasive infection. Adjuvants that can provide a more balanced Th1/Th2-type response may be required to optimize protection of GAS vaccines, particularly those based on non-M protein antigens.

## INTRODUCTION

Group A *Streptococcus* (GAS) is a bacterial pathogen responsible for a wide spectrum of clinical manifestations. Mild common infections include pharyngitis and impetigo, while more-serious infections such as streptococcal toxic shock syndrome, necrotizing fasciitis, and sepsis are relatively rare and yet life-threatening conditions. Poststreptococcal sequelae can manifest following repeated mild infections in the form of acute rheumatic fever (ARF) and rheumatic heart disease (RHD). The global burden of streptococcal diseases has been long neglected despite being responsible for an estimated 500,000 deaths annually (1). Recent advocacy efforts have highlighted the urgent need for the development of a vaccine that prevents GAS-related infections (2–4). M protein-based vaccines have been considered strong candidates, despite concerns over insufficient serotype coverage (N-terminal-based vaccines) and the association of M protein with the generation of cross-reactive antibodies linked to ARF and RHD (5). On the other hand, non-M protein-based vaccines have emerged as alternative candidates that overcome such concerns. Regardless of the choice of GAS antigen used as a vaccine candidate, the choice of adjuvant, a significant element of the final vaccine formulation, has generally been overlooked. Aluminum salts (alum) have represented the most common adjuvant used to test GAS vaccine candidates (6–9). For M-protein based vaccines, alum has proven effective for the generation of opsonizing antibodies, which are associated with protection against infection in animal models (10, 11).

However, less is known about potential correlates of protection for non-M protein-based vaccines and the role that different adjuvants may play in the protection afforded by these vaccines. Our research has focused on the development of Combo5 vaccine, a combination of 5 GAS protein antigens (arginine deiminase, [ADI], C5a peptidase [SCPA], streptolysin O [SLO], interleukin-8 protease [SpyCEP], and trigger factor [TF]). We previously tested Combo5 formulated with alum in murine and nonhuman primate (NHP) models of infection with various results. While immunization with Combo5/alum decreased the severity of clinical signs but did not reduce colonization in a NHP pharyngitis model of infection (12) and provided protection in a murine superficial skin infection model, the same formulation failed to protect in a murine invasive model of disease (13). In this study, taking advantage of the opportunity presented by these findings, we examined the protective capacity of Combo5 formulated with a panel of different adjuvants (Table 1) using the invasive GAS disease model. The level of protection from lethal challenge ranged from 20% to 90% survival among the different formulations. We further characterized the protective immune response elicited by Combo5 formulated with SMQ adjuvant and compared this to the nonprotective response elicited by Combo5/alum and to the protective response elicited by the “gold standard” homologous M1 protein formulated with alum.

**TABLE 1:** Adjuvants used in this study

Adjuvant	Composition^*a*^
Alum	Aluminum hydroxide gel (Alhydrogel 2%)
Advax-2	Inulin and CpG
Advax-4	Inulin, CpG, and murabutide
SWE	Squalene-in-water emulsion
LQ	Neutral liposomes containing DOPC, cholesterol, and QS21
LMQ	Neutral liposomes containing DOPC, cholesterol, 3D-MPL, and QS21
SMQ	Squalene-in-water emulsion containing cholesterol, 3D-MPL, and QS21

a Abbreviations: DOPC, dioleoylphosphatidylcholine; 3D-MPL, 3-*O*-desacyl-4′-monophosphoryl lipid A.

## RESULTS

### Antigen-specific antibody responses.

**FIG 1:**
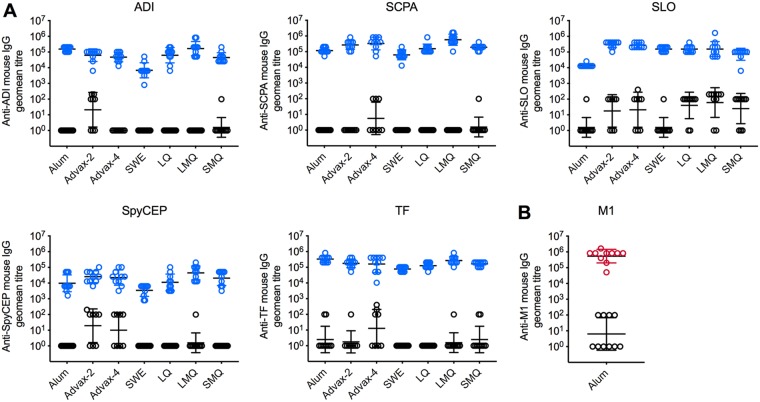
GAS antigen-specific IgG response in mouse serum. (A) Antibody titers against ADI, SCPA, SLO, SpyCEP, and TF at day 35 were significantly higher for all antigens (*P < *0.0001) in mice immunized with Combo5 (blue circles) compared to PBS (black circles) for each adjuvant tested. Adjuvants used for immunization are indicated on the *x* axes. (B) Antibody titers against M1 protein in alum (red circles) were significantly higher than those seen with the PBS controls (black circles) at day 35 (*P < *0.0001). Statistical comparisons were made using a two-tailed Mann-Whitney U test (*, *P < *0.05). Bars represent geometric mean titers ± geometric standard deviations (SD).

Groups of humanized plasminogen mice were immunized with Combo5 formulated with alum, Advax-2, Advax-4, SWE, LQ, LMQ, or SMQ adjuvant (Table 1), while negative-control groups were immunized with phosphate-buffered saline (PBS) plus the corresponding adjuvant. One primary and two booster immunizations were delivered via intramuscular injection into the thigh muscle. Serum samples from day 35 were used to measure antigen-specific antibodies against all proteins contained in Combo5. Antigen-specific total IgG endpoint titers against all antigens (ADI, SCPA, SLO, SpyCEP, and TF) with all adjuvanted vaccines (Fig. 1A, blue circles) were significantly higher than the titers seen with their corresponding negative controls (Fig. 1A, black circles). Immunization with M1/alum was used as positive control in all experiments, and Fig. 1B shows anti-M1 IgG titers from one representative experiment.

### Survival against invasive challenge.

**FIG 2:**
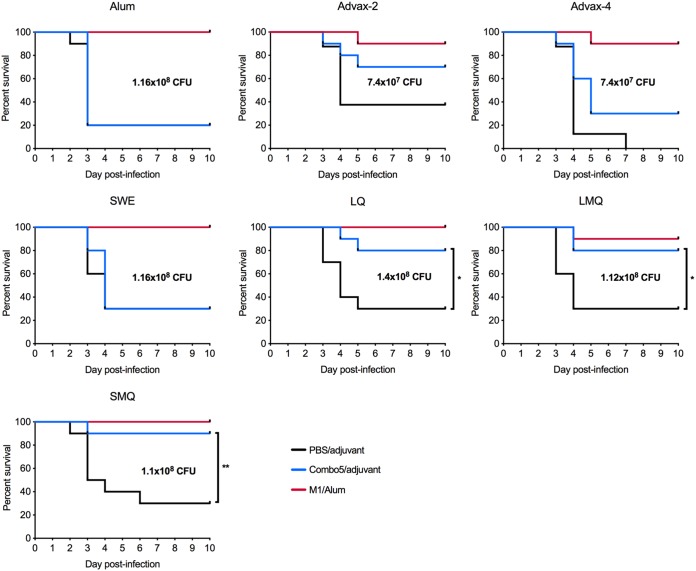
Adjuvants significantly influence protection against invasive GAS challenge. (A) Ten humanized plasminogen *Alb*PLG1 mice per group were immunized with Combo5 (blue lines) formulated in alum, Advax-2, Advax-4, SWE, LQ, LMQ, or SMQ adjuvants. (B) Mice received PBS with each corresponding adjuvant as a negative control (black lines) and M1 protein in alum as a positive control (red lines). Mice were infected on day 42 with GAS strain 5448. Infection doses for each experiment are indicated in each graph. Curves were compared using the log rank Mantel-Cox test (*, *P < *0.05; **, *P < *0.01).

On day 42, mice were infected subcutaneously with GAS strain 5448 (dose range, 7.4 × 10^7^ CFU to 1.4 × 10^8^ CFU). Mice were monitored for 10 days postinfection, and animals showing signs of disease, pain, or distress were humanely euthanized and classified as having succumbed to infection. As shown in a previous study (13), immunization with Combo5/alum did not provide significantly greater protection than treatment with PBS/alum (Fig. 2). Mice immunized with Combo5/SWE or Combo5/Advax-4 showed no protection compared to mice immunized with adjuvant alone, mirroring the situation for Combo5/alum (Fig. 2). Mice immunized with Combo5 formulated with Advax-2 showed 70% survival against lethal challenge, although the results were not significant compared to the 40% survival rate seen with mice immunized with Advax-2 alone (Fig. 2). Combo5 formulated with LQ, LMQ, and SMQ adjuvants showed significantly higher levels of survival, with 80%, 80%, and 90% protection, respectively, than that observed for controls treated with adjuvant alone (Fig. 2). Mice immunized with M1/alum were consistently protected against infection, with 90% to 100% survival across the experiments (Fig. 2).

### Characterization of protective immune response using SMQ adjuvant.

**FIG 3:**
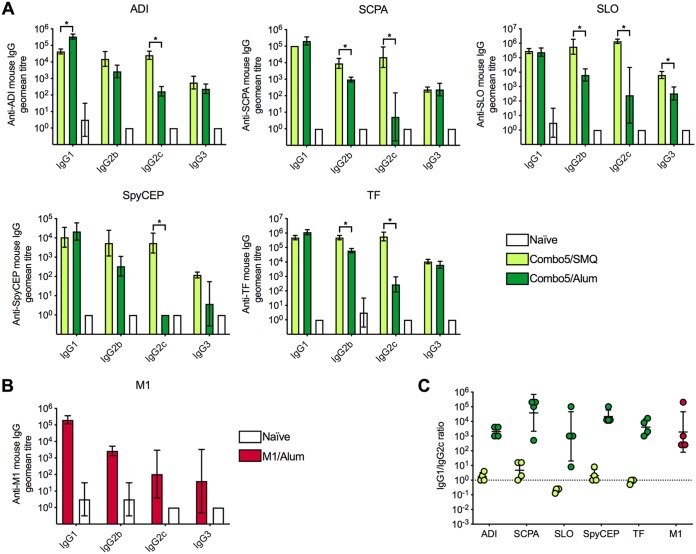
IgG subclasses generated by SMQ and alum. Naïve mice or mice immunized with Combo5/SMQ, Combo5/alum or M1/alum (*n* = 4 per group) were euthanized on day 35. Serum samples were used to measure levels of IgG1, IgG2b, IgG2c, and IgG3 (*x* axes) specific for ADI, SCPA, SLO, SpyCEP, and TF (A) and for M1 protein (B) as indicated. (C) IgG1/IgG2c ratios for each antigen. Antigen-specific titers generated by different adjuvants were compared using a two-tailed Mann-Whitney U test (*, *P < *0.05). Bars represent geometric mean titers ± geometric SD.

We further characterized the immune response induced by the formulation that provided the highest protection against invasive lethal challenge and compared it to the immune response induced by Combo5/alum and M1/alum. Four mice per group were immunized with Combo5/SMQ, Combo5/alum, and M1/alum, and naive mice were used as negative controls. At day 35 following one primary immunization and two booster immunizations, animals were humanely euthanized to obtain blood and spleens for further analyses. Levels of antigen-specific IgG subclasses (IgG1, IgG2b, IgG2c, and IgG3) were measured in serum samples. IgG1 serum titers against ADI were significantly higher in Combo5/alum-immunized mice than in those immunized with Combo5/SMQ (Fig. 3A). On the other hand, anti-ADI IgG2c titers were significantly higher in Combo5/SMQ-immunized mice than in Combo5/alum-immunized mice. Titers for anti-ADI IgG2b and IgG3 were comparable in the two groups. Anti-SCPA and anti-TF IgG2b and IgG2c titers were significantly higher in mice that received the Combo5/SMQ formulation than in those that received Combo5/alum, while titers against the same antigens were comparable for IgG1 and IgG3 (Fig. 3A). IgG2b, IgG2c, and IgG3 titers against SLO were significantly higher in Combo5/SMQ-immunized mice than in those treated with Combo5/alum, whereas the IgG1 titers were equivalent for the two formulations (Fig. 3A). Anti-SpyCEP IgG1, IgG2b, and IgG3 antibody titers were not significantly different between Combo5 formulations, but a significant difference was observed in IgG2c titers, which were between 10^3^ and 10^4^ in the Combo5/SMQ group, while the IgG2c titers were undetectable in the Combo5/alum group (Fig. 3A). The anti-M1 IgG subclass response following immunization with M1/alum was characterized by high IgG1 titers followed by IgG2b titers and low titers for IgG2c and IgG3 antibodies (Fig. 3B). IgG1/IgG2c ratios were calculated for each antigen in both Combo5 and M1 protein formulations (Fig. 3C). Ratios closer to 1 were observed for Combo5/SMQ, indicating a more balanced Th1/Th2 response (Fig. 3C). On the other hand, higher ratios were obtained for Combo5 and M1 antigens formulated with alum, suggesting a Th2-biased antibody response (Fig. 3C).

**FIG 4:**
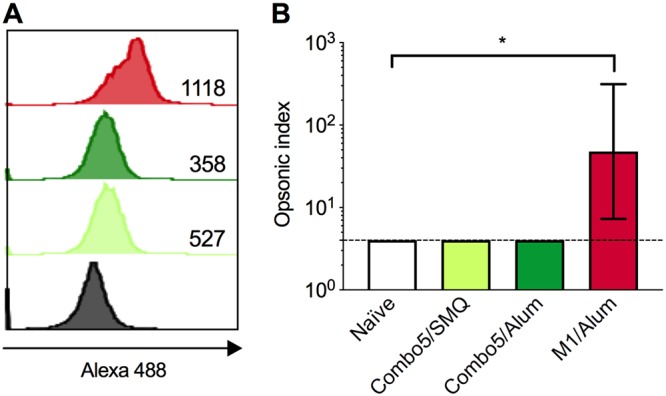
Antibodies binding to the surface of live GAS and opsonophagocytic activity. (A) Pooled serum from naive (black line) and Combo5/SMQ (light green line)-, Combo5/alum (dark green line)-, and M1 (red line)-immunized mice was incubated with live M1 5448 GAS. Binding was detected by flow cytometry, and *T*(*X*) values, shown on the lower right side of each histogram, were determined by using the probability binning algorithm in FlowJo to compare samples of bacteria incubated with immune sera to samples incubated with naive sera. A cutoff value for the statistic *T*(*X*) was established by comparing *T*(*X*) values between sample replicates, and a *T*(*X*) value of >200 was considered significant (*P* < 0.01). (B) Antibody functionality of naive mouse sera (clear bar) or Combo5/SMQ (light green bar), Combo5/alum (dark green bar), and M1/alum (red bars) immune mouse sera was tested using a standardized *in vitro* opsonophagocytic assay. Differentiated HL-60 cells were incubated in the presence of GAS M1 5448, a source of complement, and mouse sera. The opsonic index was calculated as the serum dilution where 50% killing of bacteria occurred. Opsonic indices were compared using the Kruskal-Wallis test corrected for multiple comparisons using Dunnett’s test, with *P < *0.05 considered statistically significant. Bars represent geometric means of opsonic indices ± geometric SD.

Binding of immune serum to live GAS M1T1 5448 was assessed using flow cytometry. Sera from Combo5/SMQ-immunized, Combo5/alum-immunized, and M1/alum-immunized mice showed significantly higher fluorescence than sera from naive mice as expressed by calculated *T*(*X*) values (Fig. 4A). Functionality of antibodies in sera was evaluated using an *in vitro* opsonophagocytic killing assay. Only M1 immune sera was able to promote opsonophagocytic killing of GAS (Fig. 4B) with an opsonic index significantly higher than that measured for naive mice.

**FIG 5:**
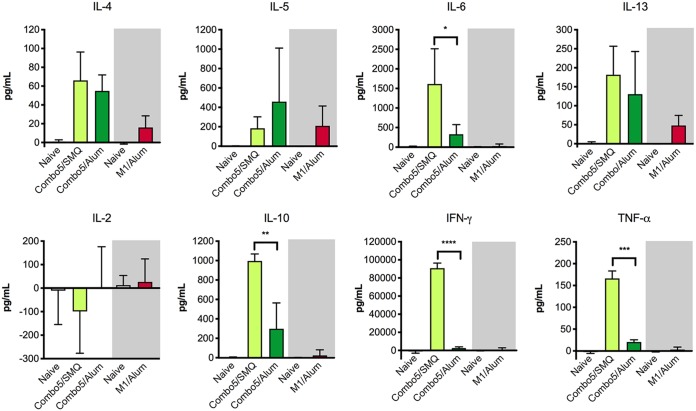
Splenocyte cytokine response to antigen stimulation. Splenocytes from naive mice or mice immunized with Combo5/SMQ or Combo5/alum (*n* = 4 per group) were stimulated with Combo5 antigens for 72 h (light area). Splenocytes from naive mice or mice immunized with M1/alum (*n* = 4 per group) were stimulated with M1 protein for 72 h (shaded area). Culture supernatants were analyzed for the presence of cytokines IL-4, IL-5, IL-6, IL-13, IL-2, IL-10, IFN-γ, and TNF-α. Bars represent mean concentrations ± geometric SD. Cytokine concentrations secreted by Combo5/SMQ and Combo5/alum immune splenocytes were compared using a two-tailed unpaired *t* test (*, *P < *0.05; **, *P < *0.01; ***, *P < *0.001).

Finally, we investigated cytokine secretion by splenocytes from immunized mice following antigen stimulation. Combo5/SMQ-immunized and Combo5/alum-immunized mice splenocytes were stimulated with Combo5 antigens, M1/alum-immunized mice splenocytes were stimulated with M1 protein, and naive splenocytes were stimulated with both Combo5 and M1 protein. Following 72 h after stimulation, culture supernatants were used to quantify a panel of 8 cytokines (interleukin-4 [IL-4], IL-5, IL-6, IL-13, IL-2, IL-10, gamma interferon [IFN-γ], and tumor necrosis factor alpha TNF-α]). Anti-M1/alum responses were characterized by secretion of IL-4, IL-5, and IL-13 (Fig. 5). Splenocytes from Combo5/alum-immunized mice secreted IL-4, IL-5, IL-8, IL-13, and IL-10 and low levels of TNF-α (Fig. 5). Splenocytes from the Combo5/SMQ group secreted IL-4, IL-5, Il-8, and IL-13 and high levels of IL-10, IFN-γ, and TNF-α (Fig. 5). A comparison between the cytokine concentrations secreted by splenocytes from Combo5/alum and Combo5/SMQ immune mice showed that the SMQ-adjuvanted formulation induced significantly higher secretion of IL-6, IL-10, IFN-γ, and TNF-α than the alum-adjuvanted formulation.

## DISCUSSION

The licensure of a GAS vaccine would bring global public health benefits. Prevention of a number of clinical manifestations caused by GAS would not only lower disease burden but also reduce the prescription of antibiotics and therefore positively contribute to the fight against antimicrobial resistance (14, 15). The non-M protein Combo5 vaccine contains 5 conserved GAS proteins (ADI, SCPA, SLO, SpyCEP, and TF) that have low sequence variation and high coverage among GAS isolates from different geographical regions (16). Using alum as an adjuvant, we have tested Combo5 in different animal models with various results. Immunization with Combo5/alum provided protection in a superficial skin infection model in mice (13) and decreased the severity of pharyngitis and tonsillitis clinical signs in a pharyngitis model in nonhuman primates (12). On the other hand, the same formulation failed to provide protection using the humanized plasminogen mouse model of invasive lethal infection (13). Given that the humanized plasminogen mouse model is a well-established model to evaluate systemic GAS dissemination (17, 18) and that vaccine-induced protection was evident in this model when purified M1 protein was used as an antigen, we decided to investigate whether alternative adjuvants could improve the efficacy of Combo5. In this study, we tested six different adjuvants (Table 1), each containing molecules with immunomodulatory properties.

Advax-2 contains delta inulin, a plant-based polysaccharide that has been shown to enhance antibody titers and cellular immune responses (19), and CpG, a TLR9 agonist that provides additional immunostimulatory properties (20). In addition to delta inulin and CpG, Advax-4 contains murabutide, which stimulates the NOD2 pattern recognition receptor (21). Both of these inulin-based adjuvants induced significantly higher antibody titers for all Combo5 antigens than were seen with PBS/adjuvant. Both Advax-2 and Advax-4 induced high antigen-specific antibody titers; however, the rate of survival of vaccinated mice was not significantly higher than that seen with the PBS-adjuvant control groups. SWE adjuvant is a squalene-in-water emulsion. The composition of SWE adjuvant is similar to that of MF59, an adjuvant currently used in several influenza vaccines available on the market (22), which has been shown to promote recruitment of immune cells to the injection site and enhance antigen uptake by antigen-presenting cells (23) by promoting local release of ATP (24). Combo5 in formulation with SWE generated high antibody titers against all antigens but showed no protection against GAS lethal challenge compared to PBS formulated with SWE.

LQ and LMQ adjuvants are neutral liposome-based formulations containing cholesterol, 1,2-dioleoyl-sn-glycero-3-phosphocholine (DOPC), and QS21, a proinflammatory saponin adjuvant that activates dendritic cells (DCs) and promotes the secretion of Th1 cytokines (25). LMQ also contains the synthetic Toll-like receptor 4 (TLR4) agonist 3-0-desacyl-4-monophosphoryl lipid A (3D-MPL). A bacterium-derived TLR4 agonist has shown a synergistic adjuvant effect when combined with the saponin QS21 in the neutral liposome carrier AS01 adjuvant. AS01 is included in Mosquirix (RTS,S) malaria vaccine and Shingrix (HZ/su) herpes zoster vaccine, both of which are approved for human use (26). SMQ is a squalene-in-water emulsion containing cholesterol, synthetic 3D-MPL, and QS21. The AS02 adjuvant, also a combination of emulsion containing a bacterial TLR4 agonist and QS21, has been tested in humans as part of the development of the Mosquirix (RTS,S) vaccine (27). Combo5 formulated with LQ, LMQ, or SMQ provided significant protection against invasive lethal GAS infection, with 80%, 80%, or 90% survival, respectively. Given that the common ingredient between these three adjuvant formulations is QS21 (Table 1), we hypothesize that addition of QS21 is crucial for protection against GAS in this invasive challenge model. Given that the antibody titers against all five proteins in Combo5 did not predict protection in the different adjuvant groups, we hypothesize that T cell immunity may be a more relevant predictor of protection in this model.

We next characterized the immune response phenotype induced by one of these protective adjuvanted formulations. SMQ was chosen as it was associated with the highest rate of survival after GAS challenge. Mice immunized with Combo5/SMQ, Combo5/alum, and M1/alum were used to study signature immune responses. IgG subclasses generated with the different formulations showed significant differences, particularly in the ability to generate antigen-specific IgG2c. Overall, immunization with Combo5/SMQ generated a combined Th1/Th2 response with more-balanced IgG1/IgG2c ratios, while Combo5/alum-adjuvanted and M1/alum-adjuvanted formulations produced a Th2-biased response (28). Serum antibodies generated by all three formulations were able to recognize and bind antigens on the surface of live GAS as measured by flow cytometry. However, only M1/alum-immunized sera were able to promote opsonophagocytosis of GAS.

A pressing issue in GAS vaccine development is the establishment of a correlate of protection that can be used to fast-track the licensure of a vaccine candidate (3). Opsonizing antibodies have long been associated with protection against infection for M protein-based vaccines and following natural infection (29, 30). In our previous report, opsonizing antibodies were detected in sera from Combo5/alum-immunized mice by the use of the classical Lancefield whole-blood indirect bactericidal assay (13). In the present study, we were not able to detect opsonic antibodies by the use of the standardized HL-60 assay for Combo5/alum. Discrepancies between these two assays in the detection of opsonic antibodies have been reported in the past (31). The technical complexity of the classical Lancefield whole-blood assay makes it unfeasible for use in clinical trials, while the HL-60 assay may become more widely accepted as the standardized assay to investigate the presence of opsonizing antibodies against GAS. In this study, mice immunized with Combo5/SMQ were protected against invasive lethal challenge in the absence of opsonizing antibodies. Consequently, this work provides evidence that the opsonizing antibody correlates of protection paradigm do not necessarily apply for non-M protein antigens. This finding highlights one of the weaknesses that need to be addressed on the road to licensure for a GAS vaccine. To begin to address this issue, cellular recall responses were investigated in antigen-stimulated splenocytes from immunized mice. Combo5/alum-immune splenocytes mainly secreted cytokines (IL-4, IL-5, IL-6, IL-10, and IL-13), indicating a Th2-type signature, with low levels of TNF-α. In contrast, Combo5/SMQ-immune splenocytes secreted a wider variety of cytokines, including Th1-type and Th2-type cytokines. Combo5/SMQ immunization resulted in strong secretion of IL-6 and IL-10 and of Th1-type cytokines IFN-γ and TNF-α, suggesting a potential role for Th1 responses in protection against invasive GAS infection following vaccination. It has been previously reported that SCPA-specific IFN-γ responses may play an important role in age-related immunity to GAS following natural exposure in humans (32). This observation, which is in line with our results, highlights the importance of Th1 responses (especially that of IFN-γ) in protective immunity against GAS infections.

Even though mouse models do not represent the best preclinical model to mimic GAS infections, they have enabled us to investigate immune mechanisms to combat infection and identify protective vaccine candidates. Mouse models are ideal for initial preclinical testing of vaccine candidates, and in this particular case, the humanized plasminogen mouse model allowed us to investigate the effect of different adjuvants to promote the protective efficacy of Combo5. To further advance Combo5/SMQ as a vaccine candidate, it is crucial to investigate its efficacy using more-representative models of infection, such as the pharyngitis model in nonhuman primates (12) or, potentially, the experimental human challenge models that are being developed (33). This pathway of vaccine testing could ideally be used for other GAS vaccine candidates, in order to build strong evidence of vaccine efficacy against GAS infections.

This work highlights the importance of adjuvant selection in order to raise protective immune responses against invasive GAS infection. Adjuvants that can provide a more balanced Th1/Th2-type response such as SMQ may be required to optimize the protection provided by GAS vaccines, in particular, those based on non-M protein antigens.

## MATERIALS AND METHODS

### Bacterial strains and growth conditions.

For recombinant protein expression, Escherichia coli BL21 Star (DE3) was grown in Luria-Bertani medium (LB) supplemented with ampicillin (100 μg/ml). Streptococcus pyogenes M1T1 strain 5448, an invasive clinical isolate (34), was grown on horse blood agar plates and in Todd-Hewitt broth supplemented with 1% (wt/vol) yeast extract. For infection experiments, a master frozen stock was prepared as previously described (12).

### Antigen expression and purification.

M1 protein and streptococcal antigens contained in Combo5 (ADI, SCPA, SLO, SpyCEP, and TF) were expressed in E. coli BL21 Star (DE3) cells and purified by affinity chromatography as previously described (12). Protein preparations were subjected to filter sterilization before final formulation. The His tag was cleaved from Combo5 antigens for enzyme-linked immunosorbent assay (ELISA) as previously reported (12).

### Adjuvants and formulations.

The composition of adjuvants used in this study is provided (Table 1). Aluminum hydroxide gel (Alhydrogel 2%; alum) was purchased from InvivoGen (CA, USA). Advax-2 and Advax-4 adjuvants with formulations based on microparticles of delta inulin were provided by Vaxine Pty. Ltd., Adelaide, Australia (35). SWE, LQ, LMQ, and SMQ adjuvant formulations were manufactured at the Vaccine Formulation Institute. SWE adjuvant (squalene-in-water emulsion) was prepared as previously described (36). SMQ adjuvant was prepared by mixing a solution of QS21 (Desert King International, CA, USA) in PBS with squalene-in-water emulsion, containing cholesterol (Merck-Sigma C1231, USA) and the synthetic TLR4 agonist 3-0-desacyl-4-monophosphoryl lipid A (3D-MPL) also called 3D-(6-acyl) PHAD (catalog no. 699855P; Merck-Avanti, USA). SMQ adjuvant was mixed with the antigen and PBS without Ca and Mg to obtain 3D-MPL at 40 μg/ml and QS21 at 100 μg/ml. LQ adjuvant was prepared by adding a solution of QS21 (Desert King International, CA, USA) in PBS to neutral liposomes prepared by the lipid film method, using 1,2-dioleoyl-sn-glycero-3-phosphocholine (DOPC) and cholesterol as lipids and rehydration in Dulbecco's phosphate-buffered saline (DPBS) (pH 7.2) without Ca and Mg buffer followed by extrusion. LQ was mixed with the antigen and PBS without Ca and Mg to obtain QS21 at 100 μg/ml. LMQ adjuvant was prepared in the same way but with incorporation of 3D-MPL into the lipid film. The LMQ adjuvant was mixed with the antigen and PBS without Ca and Mg to obtain 3D-MPL at 40 μg/ml and QS21 at 100 μg/ml.

### Immunization and challenge.

Groups (*n* = 10; 5 females and 5 males) of mice (4 to 6 weeks old) heterozygous for the human plasminogen gene (*Alb*PLG1) (17) were immunized intramuscularly on days 0, 21, and 28 with 24 μg of Combo5 (total protein) adjuvanted with each of the adjuvants listed in Table 1 to reach a final volume of 50 μl per dose. Negative-control groups were immunized with PBS combined with the corresponding adjuvant. Purified M1 protein (30 μg) formulated with alum was used as a positive control. Serum samples were taken before immunization and on day 35. On day 42, immunized mice were infected subcutaneously with GAS (100 μl), with the infecting dose confirmed on the day of infection by enumerating CFU. Mice were monitored twice daily for 10 days and given scores based on observed clinical signs (see Table S1 in the supplemental material) (37). For antibody binding, opsonophagocytic killing, and splenocyte stimulation assays, 4 mice per group were immunized with Combo5/alum, Combo5/SMQ, or M1/alum (days 0, 21, and 28). Naïve mice were used as negative controls. On day 35, mice were euthanized, blood was obtained via heart puncture, and spleens were removed under aseptic conditions.

10.1128/mBio.00122-20.1 TABLE S1 Mouse scoring system. Download Table S1, DOCX file, 0.01 MB. Copyright © 2020 Rivera-Hernandez et al. 2020 Rivera-Hernandez et al. This content is distributed under the terms of the Creative Commons Attribution 4.0 International license.

### Antigen-specific ELISA.

Antibody titers against individual antigens were determined by ELISA as described previously (12). Antigen-specific mouse IgG antibodies were detected with horseradish peroxidase (HRP)-conjugated goat anti-mouse IgG antibody (Calbiochem) at a 1:3,000 dilution. To measure titers of different IgG subclasses, secondary HRP-goat anti-mouse IgG1, IgG2b, IgG2c, and IgG3 antibodies (Abcam) were used at a 1:40,000 dilution.

### Antibody binding to the GAS surface.

Antibody binding to the GAS surface was done as previously described (12) using GAS strain 5448. Goat anti-mouse IgG conjugated to Alexa Fluor 488 (SouthernBiotech) (1:200 dilution) was used for detection. Cells were washed with PBS and fixed in 1.5% (wt/vol) paraformaldehyde–PBS. A total of 10,000 events were acquired using a BD Accuri C6 flow cytometer (BD Biosciences).

### Opsonophagocytic killing assay.

The opsonophagocytic killing assay was performed as previously described (12, 38). Briefly, 10 μl of GAS strain 5448, washed and diluted to 120,000 CFU/ml in opsonization buffer (10% [vol/vol] defined fetal bovine serum [FBS; HyClone], 0.1% [wt/vol] gelatin [Sigma-Aldrich Company Ltd.] in Hanks’ balanced salt solution with Ca/Mg), was mixed with 20 μl sera serially diluted in opsonization buffer in a round-bottomed 96-well plate and then incubated for 30 min at 700 rpm at room temperature. A 10-μl volume of baby rabbit complement, diluted one in six in opsonization buffer, and 40 μl differentiated human promyelocytic leukemia cells (HL-60) were added to each dilution of sera at a multiplicity of infection of 500:1 (HL-60:GAS) and incubated for 90 min at 37°C with 5% CO_2_ with shaking at 700 rpm. Prior to the assay, HL-60 cells were differentiated by incubation in 0.8% dimethylformamide at 37°C with 5% CO_2_ for 5 to 6 days and diluted in opsonization buffer to 1 × 10^7^ cells/ml. Plates were then incubated on ice for 30 min, and 10 μl from each well was spotted onto Todd-Hewitt yeast agar plates (Todd-Hewitt broth supplemented with 0.5% [wt/vol] yeast extract and 1.5% [wt/vol] bacteriological agar). An overlay agar, consisting of Todd-Hewitt broth supplement with 0.75% (wt/vol) yeast extract and 0.0025% (wt/vol) 2,3,5-tetraphenyltetrazolium chloride (Sigma-Aldrich Company Ltd.), was poured over each plate. Plates were incubated overnight at 37°C with 5% CO_2_. The number of surviving CFU was then determined using a ProtoCOL3 automated colony counter (Synbiosis), and the dilution of sera resulting in 50% killing was calculated as the opsonic index.

### Splenocyte stimulation.

Aseptically removed spleens were pressed against a 70-μm-pore-size cell strainer (Corning Falcon) to obtain a single-cell suspension in PBS. Cells were washed with PBS, and red blood cells were subsequently lysed using red blood cell lysis buffer (Roche). Cells were resuspended in RPMI 1640 supplemented with 10% (vol/vol) fetal bovine serum and 0.1% (vol/vol) β-mercaptoethanol. Splenocytes (1 × 10^6^ cells) were stimulated using 1 μg of Combo5 antigen mix or M1 protein, and medium was added to negative controls (unstimulated). Splenocytes were incubated at 37°C with 5% CO_2_ for 72 h. Culture supernatants were isolated by centrifugation and stored at –80°C for cytokine quantification.

### Cytokine quantification.

Cytokines secreted into the culture supernatant were quantified using a LEGENDplex mouse 8-plex Th1/Th2 panel (BioLegend) following the manufacturer’s protocol. Samples were acquired using a BD Accuri C6 flow cytometer (BD Biosciences), and data were analyzed using LEGENDplex Data Analysis software (BioLegend v8.0). The concentration of cytokines due to antigen stimulation was calculated by subtracting the concentration of cytokines in unstimulated samples from the concentration in stimulated samples.

### Statistical analyses.

Antigen-specific endpoint titers were analyzed using the two-tailed Mann-Whitney U test, with *P* values of <0.05 considered statistically significant (GraphPad Prism 8). Murine survival curves were analyzed using the Mantel-Cox log rank test, with *P* values of <0.05 considered statistically significant (GraphPad Prism 8). Flow cytometry data were analyzed using the probability binning algorithm in FlowJo 10.4.1 (Tree Star Inc.), a cutoff value of *T*(*X*) of 200 was experimentally determined, and samples having *T*(*X*) values of >200 were considered significant (*P < *0.01) (99% confidence). Opsonic indices were compared using the Kruskal-Wallis test corrected for multiple comparisons using Dunnett’s test, with *P* values of <0.05 considered statistically significant (GraphPad Prism 8). The concentrations of cytokines secreted from splenocytes were compared using a two-tailed unpaired *t* test, with *P* values of <0.05 considered statistically significant (GraphPad Prism 8).

### Ethics approvals.

All animal procedures were conducted according to the Australian Code for the Care and Use of Animals for Scientific Purposes (39). Breeding and experimental procedures using humanized plasminogen *Alb*PLG1 mice were approved by the University of Queensland Animal Ethics Committee (SCMB/136/16/NHMRC/BREED and SCMB/140/16/NHMRC).
