# Supplementary material for: Vaccine-Induced Th1-Type Response Protects against Invasive Group A Streptococcus Infection in the Absence of Opsonizing Antibodies
Source: mBio. 2020 Mar 10;11(2):e00122-20. doi: 10.1128/mBio.00122-20 (PMC7064752; doi:10.1128/mBio.00122-20)
Supplement: TABLE S1 [file mBio.00122-20-st001.docx]

| **Supplementary table 1. Mouse Scoring System** | | |
| --- | --- | --- |
| **Indicators** | **Independent variables** | **Score^1^** |
| **Locomotion** | Walking normally | *0* |
|  | Limping, stiffness, slowed movement | *1* |
|  | Visible inflammation (swollen limbs, tail or face) | *2* |
|  | No movement | *3* |
| **Behaviour** | Normal, active and responsive to stimuli | *0* |
|  | Away from littermates | *1* |
|  | Aggressive or huddled in corner | *2* |
|  | Lack of response to external stimuli | *3* |
| **Appearance** | Normal, groomed | *0* |
|  | Ruffled fur | *1* |
|  | Animal is hunched, shivering, visibly dehydrated | *2* |
|  | Reluctant to move | *3* |
| ^1^ Mice with a score of *3* on any of the indicators or a score of *2* on two or more indicators were euthanised and considered as succumbed to infection. Mice with a score of *2* on one indicator were monitored closely to determine disease progression in subsequent check-ups. | | |
